# Reduced Reactivity of Amines against Nucleophilic Substitution via Reversible Reaction with Carbon Dioxide

**DOI:** 10.3390/molecules21010024

**Published:** 2015-12-23

**Authors:** Fiaz S. Mohammed, Christopher L. Kitchens

**Affiliations:** Department of Chemical and Biomolecular Engineering, Clemson University, Clemson, SC 29634, USA; fiaz.mohammed@chbe.gatech.edu

**Keywords:** carbamate, urea, benzophenoneimine, green chemistry

## Abstract

The reversible reaction of carbon dioxide (CO_2_) with primary amines to form alkyl-ammonium carbamates is demonstrated in this work to reduce amine reactivity against nucleophilic substitution reactions with benzophenone and phenyl isocyanate. The reversible formation of carbamates has been recently exploited for a number of unique applications including the formation of reversible ionic liquids and surfactants. For these applications, reduced reactivity of the carbamate is imperative, particularly for applications in reactions and separations. In this work, carbamate formation resulted in a 67% reduction in yield for urea synthesis and 55% reduction for imine synthesis. Furthermore, the amine reactivity can be recovered upon reversal of the carbamate reaction, demonstrating reversibility. The strong nucleophilic properties of amines often require protection/de-protection schemes during bi-functional coupling reactions. This typically requires three separate reaction steps to achieve a single transformation, which is the motivation behind Green Chemistry Principle #8: Reduce Derivatives. Based upon the reduced reactivity, there is potential to employ the reversible carbamate reaction as an alternative method for amine protection in the presence of competing reactions. For the context of this work, CO_2_ is envisioned as a green protecting agent to suppress formation of *n*-phenyl benzophenoneimine and various *n*-phenyl–*n*-alky ureas.

## 1. Introduction

**Scheme 1:**
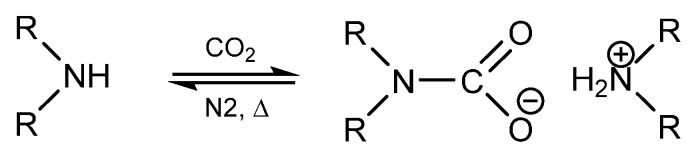
Reaction scheme of reversible alkylammonium carbamate formation from coupling of carbon dioxide and a secondary amine.

It has been previously reported that primary and secondary amines react with CO_2_ to produce thermally reversible alkylammonium alkylcarbamates as seen in Scheme 1 [1,2,3,4,5,6,7]. The CO_2_ reacts with one primary amine to form carbamic acid, which then further reacts with free amine to form the carbamate product. This mechanism has been extensively studied in literature with a variety of applications in different fields [8,9,10,11,12]. For example, the reversible amine to carbamate transition exhibits a significant change in the ionic character from non-ionic to ionic upon CO_2_ reaction, which has been employed in systems referred to as reversible ionic liquids, reversible surfactants, and switchable solvents [8,13,14,15,16,17,18,19]. These systems have included CO_2_ reaction with guanidines and amidines reaction with alcohols as two-component systems and primary or secondary amines as single component systems. In these systems, the solvent property changes can be quite appreciable, changing in character from that of hydrophilic methanol to that of hydrophobic chloroform [8,14,15]. The potential implications of such tunable solvent properties are widespread in the realm of reactions and separations, where a change in solvent properties can induce a complete phase separation and facile recovery of products, catalysts, or unreacted reagents. This was initially demonstrated by Jessop *et al.* with the reversible protonation of DBU (1,8-diazabicyclo-[5.4.0]undec-7-ene) in the presence of an alcohol and CO_2_ [8]. In the unprotonated state, the DBU/alcohol mixture is considered non-polar and is completely miscible with decane. The addition of CO_2_ induces DBU protonation and switch to a polar nature and phase separation from the non-polar decane phase. Specific potential applications that have entertained the use of switchable solvents or reversible ionic liquids have included oil extractions [20] CO_2_ capture [17,21] styrene polymerization [15,22], Claisen-Schmidt condensation, Heck reaction [13] Michael addition [13,15] cellulose acetylation [23] and nanomaterial processing [18], to name a few. In each of these applications, amine reactivity can become an impeding issue [13].

In other work, reversible surfactants use the same chemistry as the switchable solvents or reversible ionic liquids to create and break emulsions or form gels with many potential applications [15,24]. Similarly, the reversible reaction of CO_2_ with amines have been demonstrated during the development of ordered laminar materials comprised of amino-terminated silanes [25]. The formation of the hybrid material could not be possible if the absence of the carbamates. Other studies have used the reversible reaction to dramatically dictate the product selectivity during the intramolecular hydroaminomethylation of ethyl methallylic amine by reducing the nucleophilicity of the nitrogen atom [26]. Eckert and coworkers [27] used CO_2_ expanded liquids at 30 bar to increase the yield of primary amine synthesis in the hydrogenation reactions of benzonitrile and phenylacetonitrile with NiCl_2_/NaBH_4_ in ethanol. The CO_2_ induced carbamate formation prevented side reactions by precipitating the desired product from solution as a carbamate, thus simplifying the purification and increasing the yield. However, the protected carbamate was not used in any further coupling reactions. In each of these applications, CO_2_ reaction is beneficial in protecting the amine from undesired product formation.

Protecting groups provide a vital role within the field of organic synthesis, enabling the coupling of various organo-functional groups in the presence of a competing group [28]. This protection also needs to be stable under a broad range of reaction conditions and both moderately and selectively cleavable. Thus, choosing the right type of protective group is of primary significance, especially in the presence of amino containing compounds that tend to be very reactive, basic in nature, and form strong hydrogen bonds. In addition to sulfonamides and amides, carbamates are the most popular and widely used protection mechanism utilized for amino groups. Such protecting groups primarily include carbobenzoxy (CBZ), di-tertiary butoxy carbonyl (Di-t-BOC) [29], and 2-(trimethylsilyl)ethylsulfonyl) (SES) [30] groups as well as other less common groups, such as borane [31]; each having their own set of advantages and disadvantages. The t-BOC and Di-t-BOC protecting groups, for example, are utilized quite frequently in literature due to the stability during catalyzed nucleophilic substitutions and catalytic hydrogenation reactions [32]. However, de-protection requires strong acids, long reaction times and has shown to generate t-butyl cations that then require scavengers to prevent undesirable side reactions [33]. Likewise, regeneration of a CBZ protected amine is achieved by acidolysis, catalytic hydrogenation, or reduction with dissolved metals. In light of these hazards, the use of milder reagents and reaction conditions has been an area of significant potential improvement [34,35], building upon the principles of Green Chemistry. Despite the recent advancements in protection/de-protection mechanisms for amines, these traditional methods are widely lacking in both material and energy efficiency, adding to the cost and/or use of corrosive reagents.

Only recently has the concept of using CO_2_ as a simpler, greener approach to amine protection/de-protection arisen [36,37,38]. Peeters *et al.* demonstrated CO_2_ as a reversible amine-protecting agent in selective Michael additions and acylations [36]. Specifically, the reaction of primary amines was inhibited in favor of normally less reactive sulfonamides, cyclic secondary amines, or β-ketoesters for the Michael addition and complete inhibition of benzylamine acylation was achieved, effectively favoring alcohol conversion without significant byproduct. Ethier *et al.* investigated the solvent effect and addition of other bases in addition to DBU on the protection of benzylamines with CO_2_ [37]. In each case, protection of the amine was achieved in the presence of a competitive reaction and removal of the CO_2_ protecting group was achieved; however, the reactivity of the de-protected amine was not examined.

This work further explores the use of the reversible CO_2_ chemistry as a unique method of protecting amines during simple coupling reactions. All reactions were carried out with (1) the non-protected amine; (2) the carbamate analogs formed in solution via reaction with gaseous CO_2_; and (3) with the de-protected amine regenerated through a thermal treatment of the carbamate. Significant differences between this work and those of Peeters *et al.* and Ethier *et al.* are the lack of a competing reaction and measurement of reactivity following de-protection. The lack of a competing reaction is significant because it measures the decreased reactivity rather than the competitive reactivity.

## 2. Results and Discussion

### 2.1. Synthesis of n-Alkyl, n-Phenyl Urea

Urea formation via amine coupling with isocyanates has been well-studied and characterized [39,40]. The urea synthesis in this work showed 95% yield from propylamine, while other amines achieved comparable yields of 85% from hexylamine, 91% from decylamine, and 90% from octadecylamine (Table 1). Once protected in the carbamate form, the reactivity was significantly reduced for the shorter chained amines with an average decrease of 62%. After the heat treatment, de-protection of the propyl, hexyl and decyl amine was successful based on the increase in urea yield to values comparable to the non-protected amine. This demonstrates the reversibility of the carbamate protection and requirement of no pressurization or separate reaction conditions. The observed lower product yields for the de-protected amines are attributed to incomplete de-protection of the carbamates along with a loss of starting material during de-protection due to the inherent volatilities of the propyl and hexylamine. TGA showed carbamate degradation temperatures higher than their amine conjugates, thus resulting in a loss of starting material during thermal amine regeneration process (Figure S6). Decylamine, with its low volatility and ease of carbamate reversal displayed the best results, with a 93% urea yield from the de-protected amine comparable to that of the pure or un-protected amine (91%). The octadecyl carbamate showed the lowest reduction in yield and the poorest recovery in the de-protected amine reactivity. This was not an expected result as a protection/de-protection cycle had been achieved separately for the octadecyl amine. A significant difference was that the octadecyl-carbamate formation resulted in a white precipitate, however the de-protection regenerated the soluble amine analog. We hypothesize that the long alkyl chains and precipitation inhibited the complete formation of the carbamate and resulted in insufficient protection.

FTIR of the isolated ureas demonstrated high purity through the absence of carbamate or other reaction byproducts for all three scenarios (non-protected, protected and de-protected). In each FTIR spectra (Figure S1), the characteristic carbonyl (C=O) and secondary amine (N-H) absorbance associated with urea formation are consistent within all isolated products while the C-H (2800–3000 cm^−1^) absorption levels correlate very well to the alkyl chain backbone length. The three states of amine (non-protected, protected and de-protected) all produced an isolated urea product displaying similar absorbance bands (Figure S1). This indicates that the products obtained were spectrally identical and not side products due to residual carbamate presence. The urea purity was further determined by ^1^H-NMR, displaying chemical shifts characteristic to our desired products (Figures S2 and S3). The main differences in spectra were the chemical shifts at 1.3 ppm attributed to the longer alkyl backbones of hexyl and decylamine, while integration of the peaks further evidenced its structure and purity.

**Table 1:** Percentage yields of the *n*-phenyl–*n*-alkyl ureas of for pure, protected and de-protected amine.

Amine	*n*	Percentage Yield (%)		
		Non-Protected	Protected	De-Protected
Propylamine	1	95	28	85
Hexylamine	4	85	20	80
Decylamine	8	91	38	93
Octadecylamine	16	90	69	72

The urea melting temperatures (T_m_) were determined by DSC (Table 2). All measured values correlated well with literature values and possessed small melting temperature ranges, indicating high purity [41]. The n-propyl urea exhibited the highest melting point and crystallization temperature despite the shortest carbon chain, and highest volatility. Similarly, the propyl and hexyl carbamates showed an increase in the decomposition onset temperature due to the stronger charged carbamate interactions present. The ureas contain hydrogen bonding donors (NH_2_) and hydrogen bonding acceptors (C=O). The shorter alkyl chains contribute less steric hindrance and thus, a higher degree of hydrogen bonding occurs. As the chain length increases, the alkyl chain disrupts the urea-urea hydrogen bonding and order, thus reducing the melting point, as seen with n-hexyl urea (68–69 °C). As the alkyl chain increases, an expected T_m_ increase from 82 °C for decylamine to 95 °C for stearyl amine is observed, which is due to increases in energy demand for the onset of melting for higher molecular weight urea.

**Table 2:** *n*-Phenyl, *n*-alkyl urea melting points and crystallization temperatures determine from DSC.

Entry	MP (°C)	Recrystalization Temp. (°C)
*n*-phenyl, *n*-propyl urea	112–114	89
*n*-phenyl, *n*-hexyl urea	68–70	47
*n*-phenyl, *n*-decyl urea	81–83	61
*n*-phenyl, *n*-stearyl urea	95–97	84

**Figure 1:**
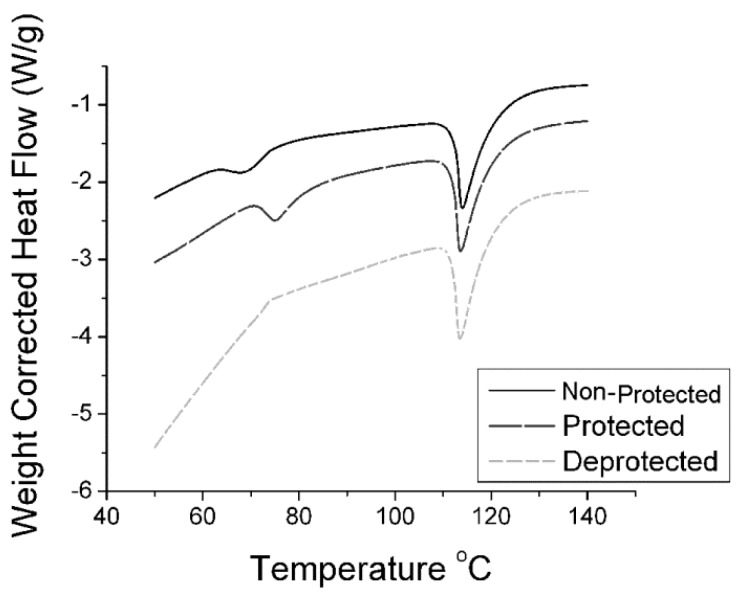
DSC spectra of n-propyl urea isolated from all three amine starting materials. Ramp rate = 5 °C per minute to 140 °C followed by equilibration to 60 °C and repeated heating.

Figure 1 displays a typical DSC curve used to determine T_m_ for each urea isolated. From the image, we can further conclude urea purity consistency throughout each amine state. As with the FTIR data, the protected and de-protected samples all provide isolated products which are chemically identical.

### 2.2. Synthesis of n-Propyl, Benzophenone(BP) Imine

**Figure 2:**
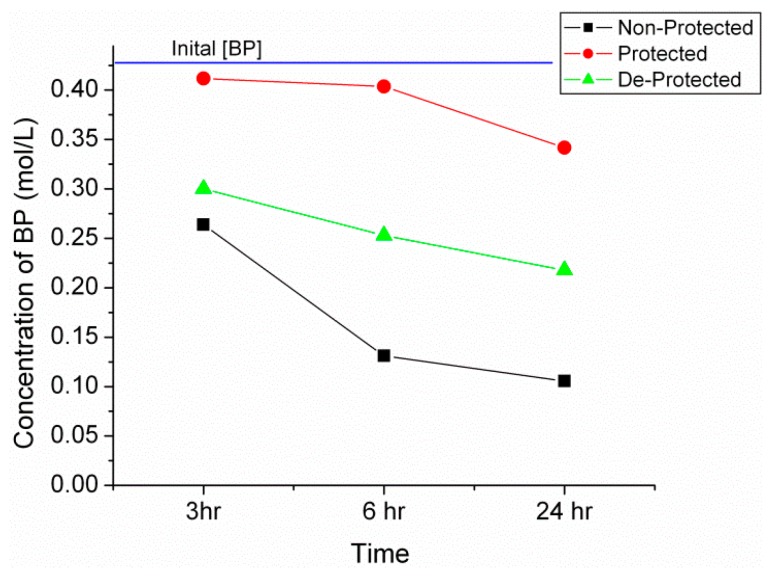
Plot of benzophenone (BP) concentration determined via gas chromatography (GC) for the non-protected, protected and de-protected propylamine reactions at 3, 6 and 24 h.

Titanium(IV) isopropoxide is a low cost mediator in BP imine synthesis [42,43,44,45,46]. According to Figure 2, the initial BP concentration of 0.45 M is slowly reduced to 0.26 M after 3 h and finally 0.11 M after 24 h as the reaction proceeds. Gas chromatography (GC) was used to determine an overall yield of 75% for the non-protected amine while a 25% yield is obtained for the protected and 50% for de-protected propylamine. Once again, a similar trend is seen with the de-protected amines falling short of the original product yield, which is attributed to the incomplete reversal of carbamates or propylamine volatilization during the de-protection process.

**Figure 3:**
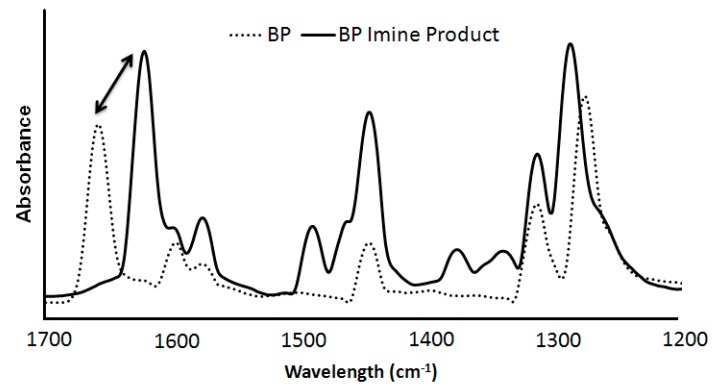
FTIR of pure BP (dashed) compared to isolated BP imine product (solid).

Although FTIR is not commonly used as a powerful quantitative tool, it was used to confirm the presence of our imine species, as well as, calculate the BP conversion. Figure 3 shows the strongly absorbing C=N shift to 1620 cm^−1^ from the C=O at 1660 cm^−1^ of the original BP [47]. This shift in absorbance demonstrates that the desired imine species was obtained during the BP-propylamine reactions and this was further evidenced via H^1^NMR and GC-MS.

The qualitative analysis was conducted over a period of 12 h, during which time ATR-FTIR spectra of aliquots of the reaction vessels were collected and analyzed to confirm the appearance of the imine product. As the reaction proceeds forward, the concentration of imine in the reaction solution will increase until equilibrium has been reached. By analyzing the absorbance ratio of C=O to C=N, the percentage of imine in the reaction vessel was determined (Table 3). These calculated values correlate very well to the percentage conversion obtained via GC and support our initial hypothesis that CO_2_ is a viable tool for the protection and inhibition of free primary amines to undergo coupling reactions.

**Table 3:** Determined BP conversion from GC analysis compared to BP conversion calculated from IR spectral C=N/C=O absorbance ratio.

Sample	Time (h)	Calculated IR Conversion (%)	Conversion from GC (%)
Non-protected	3	35	33
Non-protected	12	55	65
Protected	3	16	18
Protected	12	26	20 *

* denotes a value that was estimated using Figure S7.

In a separate set of experiments, imine formation was observed to take place without the presence of mechanical stirring (Figure S7). Here, the benzophenone, Ti catalyst and propylamine were combined in 1.5 mL GC vials and monitored *in-situ* with the absence of any a titanium complex intermediates. The percentage conversion correlated very well despite running the reaction in separate vials with maximum conversions identical to those obtained above in Figure 2. The non-protected reaction reaches a 75% equilibrium conversion after 15 h, while the protected propylamine yields a 25% equilibrium conversion after 36 h. The de-protected sample obtains an equilibrium conversion of 50% after 5 h, a phenomenon that has been consistent throughout this work. Confirmed by FTIR, a distinct reduction in CH_2_ and CH_3_ absorption provides evidence to support the loss of starting amine concentration during de-protection. An alternative explanation for the reduction in imine and urea yield using the de-protected amines is the formation of isocyanate side products during the thermal treatment [48]; however, this theory is not strongly supported.

In the presence of methanol, CO_2_ will preferentially react with the alcohol to form an alkylcarbonic acid that then reacts with the amine to form methyl carbamates, as opposed to the alkylammonium carbamates obtained under aprotic conditions [49]. The resulting carbonic structure has a larger energy requirement to cleave the methyl group and release the carbon dioxide. It is hypothesized that the reduced BP conversion for the de-protected reactions could be due to the increased difficulty to reverse the protection as the protic methanol solvent interacts with the carbamate.

Lastly, an explanation into the formation of ureas and benzophenoneimine in spite of CO_2_ induced carbamate protection needs to be discussed. For example, the 25% conversion of BP to the imine does not represent a complete protection of the propyl amine. According to Salmi *et al.* [45], the reductive amination of BP proceeds through an imine species with no observable titanium complex intermediates. Hence, the complexation of BP with the Ti is not a cause for the observed reduction in BP concentration, and this conversion is attributed to reactivity of the amine with the BP. Furthermore, control experiments in the absence of propylamine showed negligible loss in BP concentration, further supporting a residual amine reactivity under CO_2_ *vs.* complexing of BP to Ti as the source of BP conversion. We conclude that the conversion of starting material under protected conditions is primarily due to the equilibrium which exists between the carbamates and the amine (Scheme 1). As the amine is converted to urea/imine, the equilibrium can shift towards the reactants, resulting in more conversion of the starting material. This theory explains the formation of the urea/imine under the carbamate protection mechanism and an investigation into the kinetics of the carbamate reactivity can also provide useful information on the degree of protection. By fully understanding the relationship that exists between carbamates and the amine-CO_2_ system, we can calculate expected yields of the product based on the equilibrium constant of the carbamate formation.

## 3. Materials and Methods

### 3.1. General Amine Protection

The required quantity of amine was added to a round-bottom flask before the addition of any other component. A CO_2_ atmosphere was then introduced into the closed vessel via a CO_2_ gas cylinder equipped with a low pressure regulator for approximately 5 min resulting in the exothermic formation of solid white powders. The reaction solvent was then added with additional CO_2_ being bubbled through the solution for a further 3 min. The original volume of the solution was made up with pure solvent after the CO_2_ bubbling in order to preserve concentrations. The reactions with protected amines were carried out under a CO_2_ atmosphere.

### 3.2. General Amine De-Protection

Carbamate solutions of the alkylamines were de-protected using indirect heat and thermostat control with stirring. An ice-bath cooled condenser was attached to the top of the reaction vessel while a steady stream of nitrogen was introduced to aid the reversal. This was carried out for 10 min, after which fresh solvent was added to maintain the reaction concentration after nitrogen saturation. This procedure was used in attempt to convert carbamates back to the original amines. The de-protected amines were then employed in the urea/imine synthesis to ensure complete de-protection and no loss of reactivity.

### 3.3. General Characterization

An aliquot of the reaction mixture (1.5 mL) was taken from each reaction vessel and injected into an Agilent 7695A GC using the Agilent 7683B automatic liquid sampler (Agilent Technologies, Santa Clara, CA, USA). The inlet temperature was set at 300 °C with a pressure of 16 psi, the oven at 80 °C with a heating rate of 20 °C/min to 250 °C. The FID was set at 300 °C. A calibration curve for benzophenone (BP) was created by plotting the integral of the BP peak *vs.* known concentrations of BP (R_t_ = 14.5 min). Melting points were determined from DSC spectra obtained using a Thermal Analysis SDT Q600 (TA Instruments, New Castle, DE, USA) equipped with alumina pans. A steady heating rate of 5 °C/min was maintained to 350 °C with a nitrogen purge of 100 mL/min. IR spectra were collected on a Nexus 870 spectrometer (Nicolet Instrument Corporation, Madison, WI, USA) with a 4 cm^−1^ resolution using 64 scans. A fixed angle single reflection 60° hemispherical Ge crystal plate, equipped with an ATR pressure clamp, was placed in a sample compartment. The output signal was collected using a deuterated triglycine sulfate (DTGS) room temperature detector. ^1^H-NMR spectra were obtained on a Bruker 300 MHz in CDCl_3_ (Bruker Biospin Corporation, Billerica, MA, USA).

### 3.4. Synthesis of n-Alkyl, n-Phenyl Urea

**Scheme 2:**
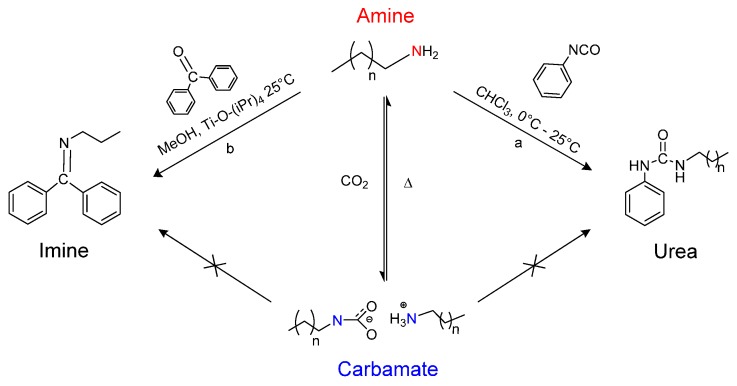
Reaction scheme showing the pathways for *n*-phenyl, *n*-alkyl urea synthesis in CHCl_3_ (**a**); and benzophenoneimine synthesis in methanol (**b**).

Exactly 50 µL (0.0545 g, 0.46 mmol) of phenyl isocyanate was added dropwise to a solution of alkylamine (0.92 mmol) in 2 mL of dry CHCl_3_ at 0 °C. The resulting solution was stirred for 60 min at room temperature and then precipitated into 25 mL of pentane. The product was isolated as a white powder via filtration and washed with several portions of pentane before being dried under vacuum at 50 °C. The products isolated from the alkylcarbamates were alternatively re-dispersed in dry CHCl_3_ and underwent thermal treatment to remove any residual carbamates. The ureas were then re-crystallized in pentane again and isolated via filtration to determine yield (Scheme 2a).

### 3.5. Synthesis of n-Propyl, Benzophenone(BP) Imine

Propylamine (5 mmol) was added to 5 mL of dry methanol, followed by 2.5 mmol benzophenone (BP) and 3.3 mmol titanium(IV)-isopropoxide. The solution was stirred under nitrogen at room temperature while aliquots were taken at 3, 6 and 24 h for GC analysis and ATR-FTIR monitoring. Alternatively, the reaction was separated into 4 GC vials and sampled *in-situ* without stirring. Each GC vial represented a reaction vessel that was used to monitor the reaction kinetics (Scheme 2b).

## 4. Conclusions

In conclusion, we have shown that the reaction of CO_2_ with alkyl amines reduced the amine reactivity resulting in reduced urea and imine yields for multiple primary amines. Performing the reaction in the absence of a competing reaction demonstrates a true decrease in reactivity, rather than preferentiality. Reversible carbamate formation via gaseous CO_2_ alone shows potential as a simple, “green” alternative compared to traditional methods that require harsh chemicals and more energy intensive reaction conditions; adhering to the major strategies of green chemistry which promote reduced solvent and energy use, inherently safer reagents, and reduced number of transformations.

Despite the novelty, the temperature dependence of the reverse reaction does introduce limitations. The ability to efficiently protect and de-protect the amine and the solubility of the carbamates in the reaction media are also potential limitations. In future work, this protection mechanism will be implemented in a competitive reaction where amine functionality is required post protection for a separate coupling reaction. This will test the limits and efficiency of the proposed protection/de-protection technique. Also, this technique will be applied to high pressure systems in an attempt to dictate and shift the carbamate equilibrium to favor the protection. The energy input to maintain higher pressures may increase, but it is offset by the reduction in solvents for conducting the three separate steps in traditional protection methods.

We anticipate that the results from this work will encourage chemists to not only design greener routes for amine protection/de-protection mechanisms, but also to learn about the many advantages that carbamate chemistry has to contribute to safer lab and industrial practices.

## Supplementary Materials

Supplementary materials can be accessed at: http://www.mdpi.com/1420-3049/21/1/24/s1.
