# Supplementary material for: Reduced Reactivity of Amines against Nucleophilic Substitution via Reversible Reaction with Carbon Dioxide
Source: Molecules. 2015 Dec 23;21(1):24. doi: 10.3390/molecules21010024 (PMC6273814; doi:10.3390/molecules21010024)
Supplement: Supplementary file 1 [file molecules-21-00024-s001.pdf]

Supplementary Materials: Reduced Reactivity of Amines against Nucleophilic Substitution via Reversible Reaction with Carbon Dioxide

Fiaz S. Mohammed and Christopher L. Kitchens *

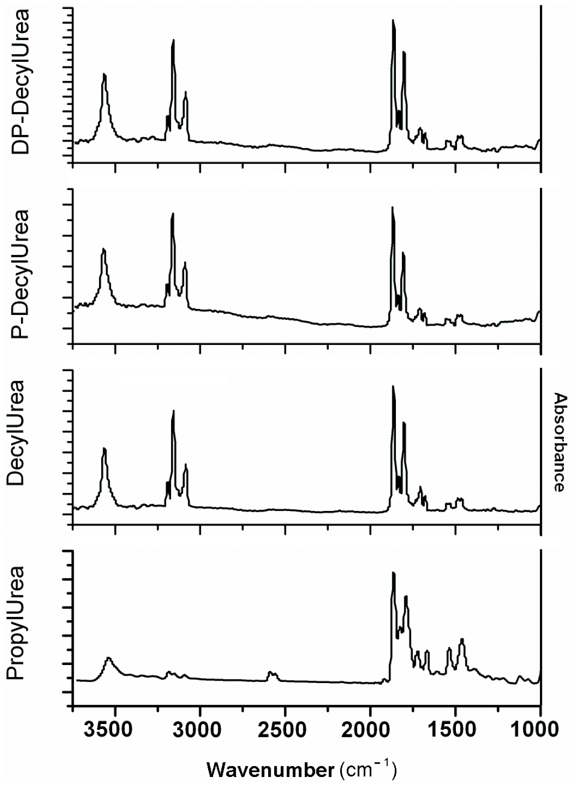

Figure S1. ATR-FTIR of *n*-phenyl, *n*-alkyl ureas showing the similar absorption spectra and characteristic carbonyl and secondary amine peak.

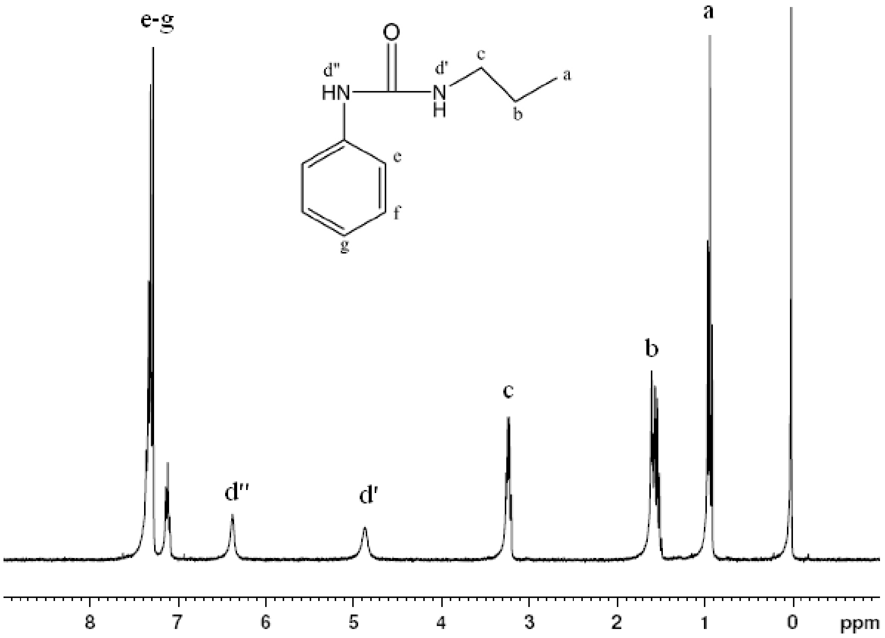

Figure S2. ^{1}H-NMR of *n*-phenyl, *n*-propyl urea. δ = 1.0 (t, 3H), 1.6 (m, 2H), 3.3 (t, 2H), 4.9 (s, 1H), 6.4 (s, 1H), 7.1–7.5 (5H).

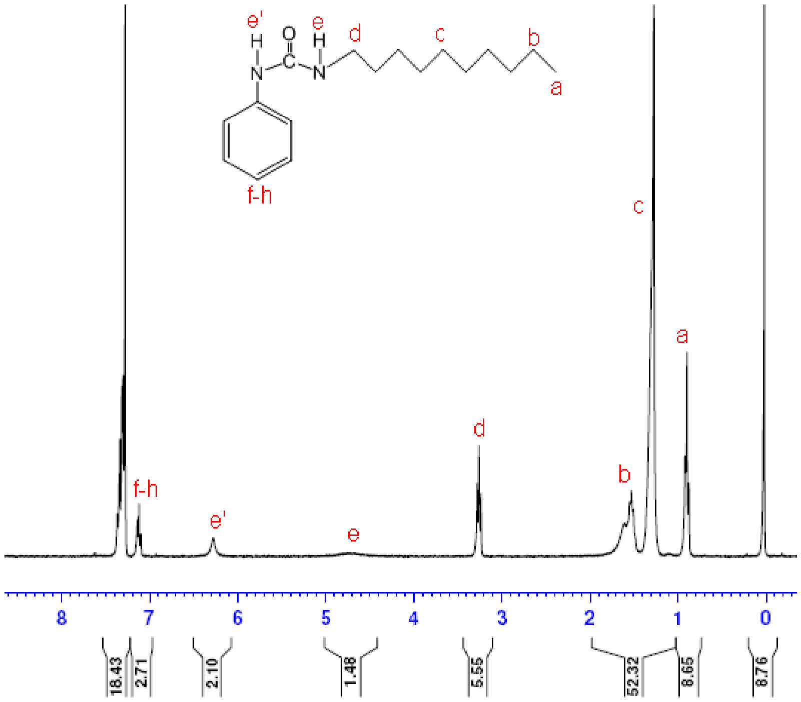

Figure S3. ^{1}H NMR of *n*-phenyl, *n*-decyl urea.

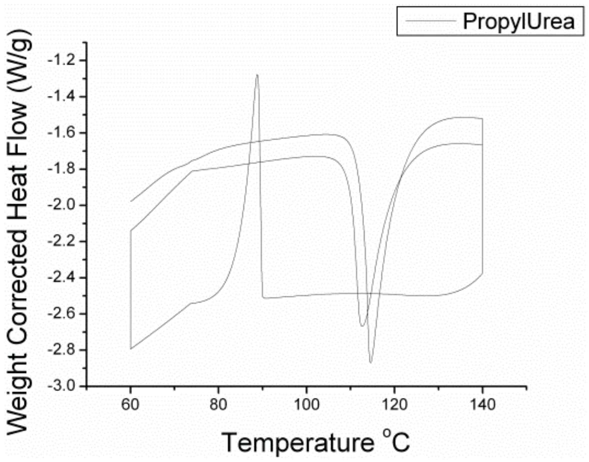

Figure S4. DSC spectra of *n*-propyl urea at ramp rate 5 °C per minute to 140 °C followed by equilibration to 60 °C and repeated heating.

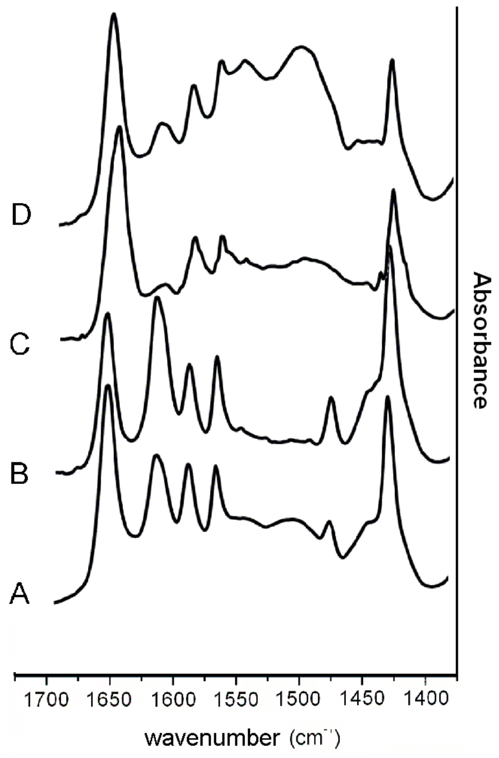

Figure S5. ATR-FTIR spectra of non-protected BP reactions at 3 h (**A**) and at 12 h (**B**). ATR-FTIR of protected BP reaction at 3 h (**C**) and at 12 h (**D**)

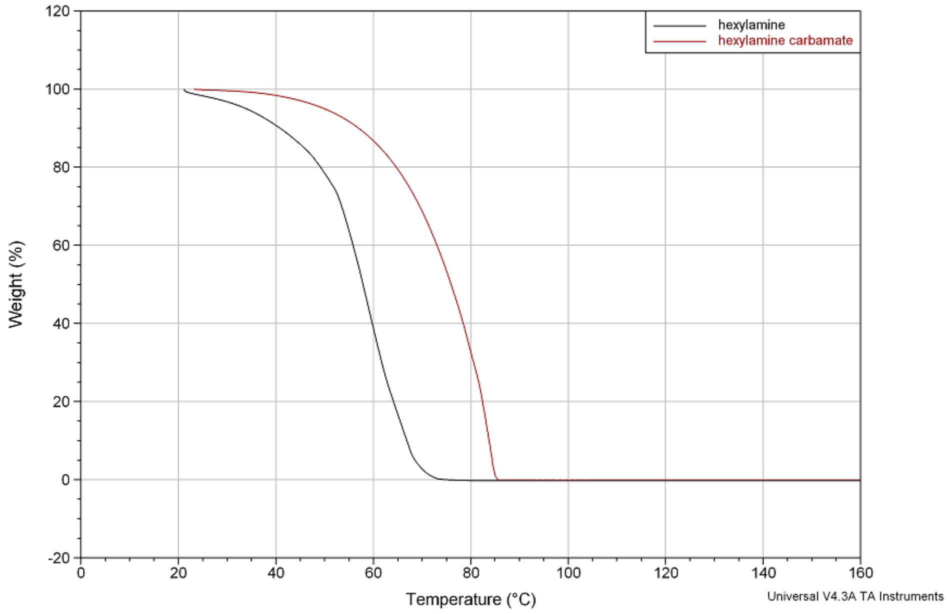

Figure S6. Thermogravametric analys is (TGA) of hexylamine and hexylamine carbamate.

The temperature at which the CO_2 evolves from neat hexylammonium carbamate powder is higher than that temperature at which the neat hexylamine will volatilize. Hence, it is very important to control the de-protection technique as some starting amine can be lost due to vaporization. This also applies to a greater extent for propyl amine which is more volatile than the hexylamine.

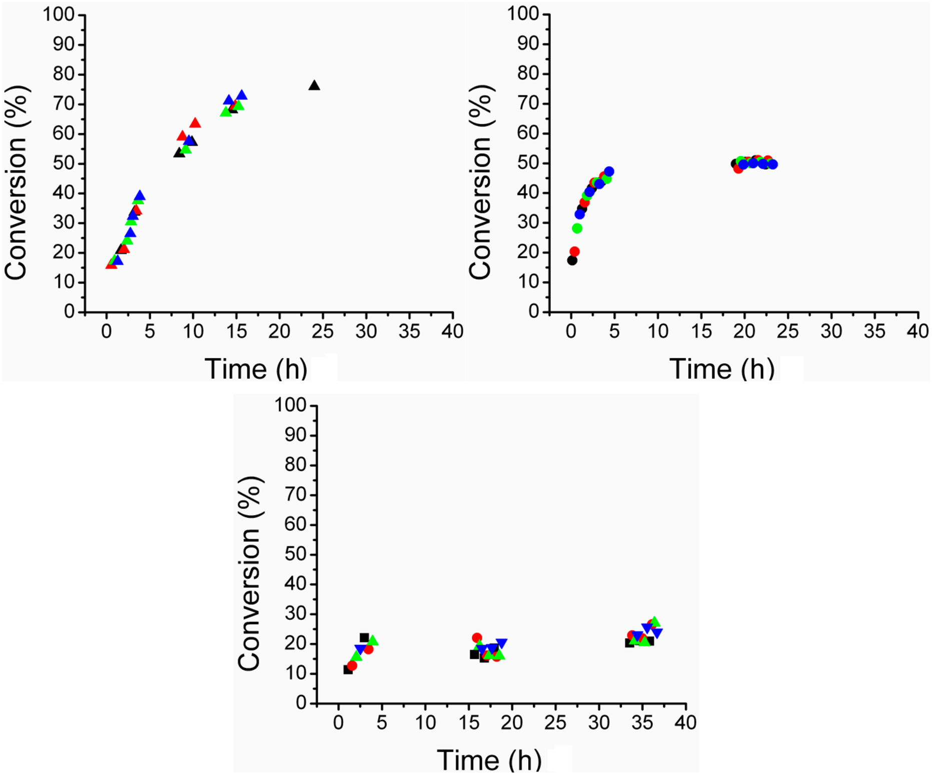

Figure S7. BP conversion % of Non-protected (**top**), Protected (**middle**) and De-protected (**bottom**) reactions monitored in-situ from 4reaction vials.
